# Novel mutations in *BBS5* highlight the importance of this gene in non-Caucasian Bardet–Biedl syndrome patients

**DOI:** 10.1002/ajmg.a.32136

**Published:** 2008-02-15

**Authors:** Tina Duelund Hjortshøj, Karen Grønskov, Alisdair R Philp, Darryl Y Nishimura, Adebowale Adeyemo, Charles N Rotimi, Val C Sheffield, Thomas Rosenberg, Karen Brøndum-Nielsen

**Affiliations:** 1 Kennedy Center, Medical Genetics LaboratoryGlostrup, Denmark; 2 Department of Paediatrics, Howard Hughes Medical Institute, University of IowaIowa City, Iowa; 3 Department of Ophthalmology, Howard Hughes Medical Institute, University of IowaIowa City, Iowa; 4 National Human Genome Center, Howard UniversityWashington, District of Columbia; 5 Kennedy Center, National Eye ClinicHellerup, Denmark

## To the Editor:

Bardet–Biedl syndrome (BBS) is a rare genetically heterogeneous disorder presenting with retinal dystrophy, postaxial polydactyly, obesity, renal malformations, learning disabilities, and male hypogenitalism [Beales et al., 1999]. To date, 12 genes have proven to be implicated in the disease, accounting for the mutational load in 70–90% of the patients [Blacque and Leroux, 2006; Stoetzel et al., 2007]. BBS is inherited as an autosomal recessive disease though in some instances, digenic triallelic inheritance has been suggested [Katsanis et al., 2001]. Although mutations in many different BBS genes have been described in Caucasians, *BBS1* and *BBS10* are the two major genes accounting for greater than 50% of the BBS patients [Mykytyn et al., 2002; Stoetzel et al., 2006a]. Mutational findings in the remaining known genes have predominately been reported in non-Caucasian patients [Ansley et al., 2003; Chiang et al., 2004; Stoetzel et al., 2006b]. *BBS5* is a minor contributor to BBS as only 2% of families from various ethnic backgrounds harbor *BBS5* mutations [Li et al., 2004].

We studied five BBS patients from two nonconsanguineous families residing in Denmark: A Somali family (Family 1) with five siblings of whom four were affected, and an affected boy from Sri Lanka (Family 2). The Sri Lankan patient was a single adopted child and no further information of his biological parents was available. The patients were identified from the files of the Retinitis Pigmentosa Registry at the National Eye Clinic, Hellerup, Denmark [Haim, 2002]. Diagnosis was based on an ERG-verified panretinal photoreceptor dystrophy in association with three or more systemic manifestations; that is, postaxial polydactyly, obesity, cognitive impairment, renal signs, and male hypogenitalism [Beales et al., 1999]. DNA from the patients was collected for mutation analysis. The control group consisted of 43 East Africans (Kenya) and 58 West Africans (Nigeria) for family 1 [Rotimi et al., 2001], and 54 Indian individuals for family 2. Appropriate informed consent was obtained from the patients and their families.

As part of a larger study screening of *BBS1*, *BBS2*, *BBS4*, *MKKS*, and *BBS10* was done by denaturing high performance liquid chromatography (DHPLC, Varian, Inc., Palo Alto, CA) followed by DNA sequencing of aberrant products on an ABI3100 automated capillary sequencer using Big Dye Terminator v.3.1 (Applied Biosystems, Foster City, CA; authors' unpublished data, manuscript submitted). *BBS5* mutational analysis was likewise performed by DHPLC. Primer sequences and PCR protocols for *BBS5* are available upon request.

Genotyping of the two families was done separately. For Family 1 we used the Affymetrix GeneChip Mapping 10K 2.0 Array to search for regions of shared genotypes among the affected siblings. Sample processing for this part of the study was carried out at the Microarray Facility in Tübingen, Germany. For Family 2, SNP genotyping was performed on Affymetrix GeneChip Human Mapping 50K Hind 240 SNP microarrays (Affymetrix, Santa Clara, CA). We allowed SNPs that could not be scored to be included in the regions of interest. An average call rate >95% was obtained.

**Fig. 1:**
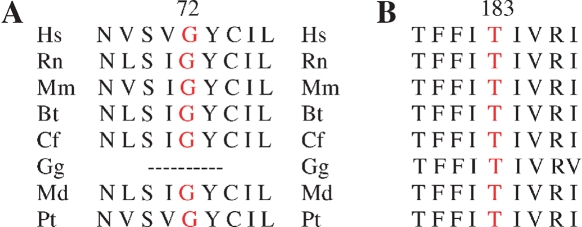
Evolutionary conservation of *BBS5* surrounding novel missense mutation sites showing local alignment of amino acid sequence. **A**: c.214G>A (p.Gly72Ser). **B**: c.574A>G (p.Thr183Ala). Hs, *Homo sapiens*; M, *Mus musculus*; Gg, *Gallus gallus*; Md, *Monodelfis domesticus*; Rn, *Rattus norvegicus*; Bt, *Bos Taurus*; Cf, *Canis familiaris*; Pt, *Pan troglodytes*.

Screening of *BBS1*, *BBS2*, *BBS4*, *MKKS*, and *BBS10* by DHPLC did not reveal causative mutations in the five patients. We therefore performed SNP genotyping in order to identify other BBS loci. Since we had no information of consanguinity in Family 1, we searched for regions of shared genotypes covering more than 5 Mb among the four affected sibs and different from the unaffected sib. We identified three major regions: Two regions at 6q15 (23 Mb) and 12q32 (34 Mb) did not contain any known BBS gene while a region spanning 11 Mb at 2q31 contained the *BBS5* locus (see the online Table I at http://www.interscience.wiley.com/jpages/1552-4825/suppmat/index.html). Mutational analysis of *BBS5* revealed a homozygous nucleotide change, c.214G>A (p.Gly72Ser) in exon 4, identified in all four affected siblings in Family 1 but not in the unaffected sib. Both parents were carriers. The mutation was absent in 202 ethnically matched control chromosomes. Sequence alignment showed the change to be localized within a conserved region (Fig. 1).

SNP genotyping of the patient in Family 2 lead to the identification of nine homozygous regions including two regions containing a known BBS gene. One locus spanning 19.6 Mb at 1p32 contained *TRIM32* (*BBS11*) while the other locus spanning 16.6 Mb at 2p31 contained *BBS5* (see the online Table I at http://www.interscience.wiley.com/jpages/1552-4825/suppmat/index.html). Sequence analysis of *TRIM32* did not reveal any causative nucleotide changes in the patient. However, direct screening of the entire *BBS5* gene identified a novel single base pair change, c.547A>G, in exon 7 in the homozygous state predicted to result in a non-conserved amino acid change, p.Thr183Ala (Fig. 1). The mutation was absent in 108 ethnically matched control chromosomes. Furthermore, it was not detected among 60 BBS patients primarily of Northern European origin (authors' unpublished data, manuscript submitted). No family members were available for testing.

**Fig. 2:**
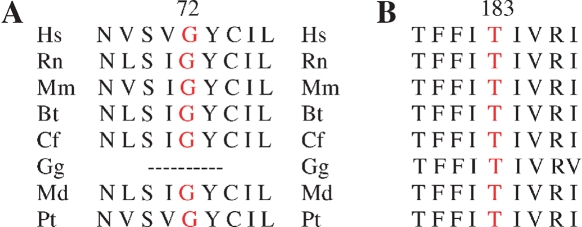
Diagram of the *BBS5* protein. Origin of exons is shown as boxes. The positions of the previously reported sequence variations and those reported here are indicated with reference to the exon where mutations occurred. Protein alterations are indicated with the one-letter abbreviations. Though the mutations mainly are truncating mutations and thereby affecting other parts than the DM16 domains, the missense mutations are all localized within the two domains. p.Asn184Ser (N184S) and p.Arg207His (R207H) are of uncertain pathogenecity due to their detection in the heterozygous state in BBS patients.

We report here on two novel missense mutations in *BBS5*. Both mutations are localized within conserved regions of the gene, are present in the homozygous state in the patients, and are absent in control chromosomes. In silico analysis predicts the mutations to affect protein function and in Family 1 the mutation segregates with the phenotype. Most of the known mutations in *BBS5* are localized within either of two putative domains, called DM16 (Fig. 2); as for the mutations reported here, p.Gly72Ser is localized in the first domain while p.Thr183Ala is localized in the second DM16 domain of *BBS5*. DM16 is a domain of unknown function and evolutionary conserved among many species [Li et al., 2004]. The fact that the mutations reported here are located in the DM16 domains supports their pathogenecity. We cannot rule out though, the existence of another mutation in linkage disequilibrium with these missense mutations. However, all coding regions plus 20 base pairs in the flanking regions were sequenced. Our findings represent the first missense mutations detected in the homozygous state in *BBS5* patients. Thus, these *BBS5* missense mutations might be useful for functional studies (Table II).

**Table II:** Mutations Reported in *BBS5*

BBS families	c.DNA	Predicted effect	Exon	State	Origin	Reference
1	c.123delA	p.Gly42GlnfsX11	2	Ho	Tunisia	Smaoui et al. 2006
1	263_271indelGCTCTTA^1^	Indel-1 fs X^1^	3	Ho	Turkey	Li et al. 2004
1	c.176G>A	p.Trp59X	3	Ho	Kurdish	Li et al. 2004
1	c.214G>A	p.Gly72Ser	4	Ho	Somalia	This study
1	c.181T>A/G	p.Leu142X	6	Ho	Saudi Arabia	Li et al. 2004
1	IVS6+3A>G^1^	fsX in exon 7^1^	7	Ho	New Foundland	Li et al. 2004
1	c.547G>A	p.Thr183Ala	7	Ho	Sri Lanka	This study
2	c.551A>G	p.Asn184Ser^2^	7	He	Caucasian	Li et al. 2004
2	c.620G>A	p.Arg207His^2^	8	He	Caucasian	Li et al. 2004
1	Deletion in intron 8→3′UTR	Exon 9–12 spliced out	9–12	Ho	Turkey	Nishimura et al. 2005

For cDNA numbering +1 corresponds to the A of the first ATG translation initiation codon, except for ^1^ where the mutations are reported as in the reference paper; ^2^ uncertain pathogeneicity; nucleotide numbers are derived from GenBank, RefSeq cDNA accession numbers: NM_152384.2 for *BBS5*.

Only five other point mutations, two indels and one large deletion have been previously reported in *BBS5* (Table II) [Li et al., 2004; Nishimura et al., 2005; Smaoui et al., 2006]. Most of these were detected in the homozygous state with the exception of the two previously reported missense variants (p.Asn184Ser and p.Arg207His). The previously published mutations are identified in patients from Africa and the Middle East, and in a single patient from New Foundland [Li et al., 2004; Smaoui et al., 2006].

In conclusion, we report two novel missense mutations in *BBS5*. Several pieces of information support that the mutations are pathogenic. Both patients are non-European. Screening of 60 patients from Northern Europe revealed no mutations in *BBS5*. These data might have implications for the mutational screening strategy of *BBS5*.

## ELECTRONIC DATABASES

Online Inheritance of Man (OMIM)—*BBS5*:# 603650.

GenBank RefSeq cDNA accession number: NM_152384.2.
